# Dynamic Functional Modulation of CD4^+^ T Cell Recall Responses Is Dependent on the Inflammatory Environment of the Secondary Stimulus

**DOI:** 10.1371/journal.ppat.1004137

**Published:** 2014-05-22

**Authors:** Chulwoo Kim, David C. Jay, Matthew A. Williams

**Affiliations:** Department of Pathology, University of Utah, Salt Lake City, Utah, United States of America; University of Pennsylvania, United States of America

## Abstract

The parameters that modulate the functional capacity of secondary Th1 effector cells are poorly understood. In this study, we employ a serial adoptive transfer model system to show that the functional differentiation and secondary memory potential of secondary CD4^+^ effector T cells are dependent on the inflammatory environment of the secondary challenge. Adoptive transfer of TCR transgenic lymphocytic choriomeningitis virus (LCMV) Glycoprotein-specific SMARTA memory cells into LCMV-immune hosts, followed by secondary challenge with *Listeria monocytogenes* recombinantly expressing a portion of the LCMV Glycoprotein (Lm-gp61), resulted in the rapid emergence of SMARTA secondary effector cells with heightened functional avidity (as measured by their ability to make IFNγ in response to *ex vivo* restimulation with decreasing concentrations of peptide), limited contraction after pathogen clearance and stable maintenance secondary memory T cell populations. In contrast, transfer of SMARTA memory cells into naïve hosts prior to secondary Lm-gp61 challenge, which resulted in a more extended infectious period, resulted in poor functional avidity, increased death during the contraction phase and poor maintenance of secondary memory T cell populations. The modulation of functional avidity during the secondary Th1 response was independent of differences in antigen load or persistence. Instead, the inflammatory environment strongly influenced the function of the secondary Th1 response, as inhibition of IL-12 or IFN-I activity respectively reduced or increased the functional avidity of secondary SMARTA effector cells following rechallenge in a naïve secondary hosts. Our findings demonstrate that secondary effector T cells exhibit inflammation-dependent differences in functional avidity and memory potential, and have direct bearing on the design of strategies aimed at boosting memory T cell responses.

## Author Summary

A key to the development of strategies for manipulating immune responses is the identification of the factors that regulate the generation of memory T cells. Many vaccination strategies rely on multiple injections to boost memory cell numbers, yet the factors that regulate the function and survival of memory T cells following multiple challenges are not fully understood. Here, we define key parameters during boosting that regulate the functional capacity and longevity of memory T cells. We report that the boosting of highly functional and long-lived memory T cells is dependent on both the activation environment and duration of the secondary challenge. Our findings demonstrate that T cells have functional plasticity that depends on the inflammatory environment of the secondary T cell activation and have direct bearing on the design of strategies aimed at generating highly functional memory T cells.

## Introduction

During acute viral and bacterial infections, antigen-specific naïve T cells clonally expand and acquire effector functions that contribute to pathogen clearance. Upon elimination of the pathogen, a small proportion of effector T cells survive and differentiate into long-lived memory cells that provide rapid and enhanced protection against secondary challenge. Activated T cells have been shown to integrate numerous signals during the primary response that impact downstream effector and memory T cell differentiation [1], [2]. Identification of signals that lead to the generation of functional memory T cells is a major goal for the design of vaccines and immunotherapies.

The transition from the effector T cell phase to the formation of memory T cells is marked by the acquisition of heightened sensitivity to low levels of antigen, often referred to as functional avidity [3]. We have recently shown that sustained interactions between the T cell receptor (TCR) and peptide antigen presented by Class II MHC (pMHCII) promote the differentiation of long-lived CD4^+^ memory T cells [4]. TCR signals also influence the survival of activated CD4^+^ T cells and the differentiation of T helper effector and regulatory subsets [5]–[11]. However, T cell extrinsic differentiation cues, including inflammatory signals such as IL-12 and IFNγ, also play a long-appreciated and critical role in driving Th1 cell differentiation. The mechanisms by which external differentiation cues control memory Th1 cell continue to be a topic of intense study, although opposing roles for the cytokines IL-2 and IL-21 in promoting effector and central memory T cell differentiation, respectively, have been reported [12]–[16].

Recent evidence indicates that external differentiation cues can influence the functional avidity of Th1 effector cells (defined as their ability to generate a functional response antigen stimulation). For example, we reported that the functional avidity of TCR transgenic Th1 effector T cells, with monoclonal antigen specificity, is not fixed, suggesting that the ability of individual T cell to translate TCR signals into a functional response can change in a TCR-independent manner [3]. Both CD8^+^ and CD4^+^ TCR transgenic T cells undergo changes to their functional avidity throughout the primary effector response to infection [17], [18], and we have previously reported that SMARTA TCR transgenic T cells, with monoclonal specificity to the LCMV-derived Class II-restricted epitope GP_61-80_, increase their functional avidity as they transition from effector to memory [3]. One possibility is that TCR-independent signals influence memory T cell differentiation in part by modulating the ability of T cells to incorporate TCR signals in response to antigen.

While many studies have focused on changes to T cell functional avidity during the primary effector and memory T cell response to infection, less is known about the mechanisms that control T cell function and secondary memory differentiation following secondary challenge. For CD8^+^ T cells, repetitive reactivation of memory T cells resulted in the acquisition of more effector-like phenotype [19], a differentiation status associated with enhanced protection from some infections [20] but not others [21]–[23]. Additionally, infection-induced inflammatory signals such as IL-12 have also been shown to enhance the functional avidity (defined as the ability to make functional responses to antigen stimulation) of secondary effector CTL [24], [25]. As compared to primary memory CD8^+^ T cells, secondary CD8^+^ memory T cells exhibit enhanced cytolytic capabilities and provide enhanced protection against certain infections such as *Listeria monocytogenes*, whereas they are more prone to functional exhaustion following chronic antigen exposure [26], [27]. Therefore, one can conclude that the functional characteristics of secondary effector CTL depend at least in part on the nature of the secondary stimulus.

Both naïve and memory CD4^+^ T cells show a similar delay in the onset of cell proliferation after exposure to antigen, despite the fact that memory T cells become activated and produce effector cytokines within several hours [28]. In the context of influenza A virus, secondary effector CD4^+^ T cells display distinct functional and phenotypic characteristics as compared to primary CD4^+^ effector T cells, including enrichment for producers of multiple cytokines, enhanced trafficking to tissue sites of infection and greater contribution to viral clearance [29]. The strength of pathogen rechallenge may also play a key role in mediating changes to the long-term fate and function of secondary Th1 cells [30]. We previously found that, unlike CD8^+^ memory T cells, a homologous secondary challenge failed to induce robust secondary expansion of CD4^+^ memory T cells [30]. A rapidly cleared homologous rechallenge of lymphocytic choriomeningitis virus (LCMV)- or *Listeria monocytogenes* (Lm)-immune mice, resulted in poor functional avidity of secondary Th1 effector function and decreased survival of secondary CD4^+^ memory T cells, Conversely, reciprocal heterologous rechallenge with a pathogen sharing single CD4^+^ T cell epitope resulted in Th1 secondary effector cells with high functional avidity and stable maintenance of secondary CD4^+^ memory T cells [31]. Certain aspects of secondary Th1 effector cell function are dependent on the pathogen used for the secondary challenge, providing additional evidence of a role for infectious environment in the differentiation of secondary Th1 effector and memory cells [31], [32].

In an effort to further define the environmental differentiation cues that regulate the function of secondary Th1 effector cells, we employed a serial adoptive transfer system that allowed us to manipulate the stimulatory environment of the recall response. Initially, we injected naïve mice with small numbers of T cell receptor (TCR) transgenic SMARTA CD4^+^ T cells (specific for LCMV Glycoprotein) and infected with LCMV one day later. Following pathogen clearance and the establishment of memory (>42 days post-infection), SMARTA memory cells were isolated and parked in either infection-matched LCMV-immune or naïve uninfected secondary hosts. Memory SMARTA cells were then induced to undergo a recall response following infection with *Listeria monocytogenes* expressing the MHC Class II-restricted LCMV GP_61-80_ epitope (Lm-gp61). As compared to SMARTA recall responses in LCMV-immune secondary hosts, SMARTA recall responses in naïve secondary hosts resulted in secondary effector cells with poor functional avidity (as measured by IFNγ production following *ex vivo* restimulation with decreasing concentrations of GP_61-80_ peptide), increased death during T cell contraction following pathogen clearance and poor maintenance of the resulting secondary Th1 memory cells.

The decrease in SMARTA cell functional following recall responses in naïve secondary hosts occurred in the later stages of the secondary response, and these hosts also exhibited a higher pathogen load. While transfer of higher numbers of SMARTA memory cells prior to rechallenge led to decreased expansion due to clonal competition for antigen, it did not prevent their loss of functional avidity, suggesting that environmental differences in inflammatory milieu induced by Lm-gp61 challenge of either naïve or LCMV-immune hosts, rather than differences in access to antigen, induced the acquisition or loss or functional avidity. In support of this, *in vivo* neutralization of IL-12, exacerbated the loss of functional avidity by SMARTA recall responses in naïve secondary hosts, whereas disruption of IFN-I activity resulted in enhanced functional avidity. Loss of functional avidity corresponded to defects in TCR signaling events, leading us to conclude that TCR-independent inflammatory cues can regulate TCR-mediated activation and differentiation signals. Our findings define key parameters that regulate the acquisition of secondary CD4^+^ effector T cell function and the formation of stable secondary memory following rechallenge.

## Results

### Functional avidity of secondary Th1 effector cells depends on the secondary stimulus

**Figure 1:**
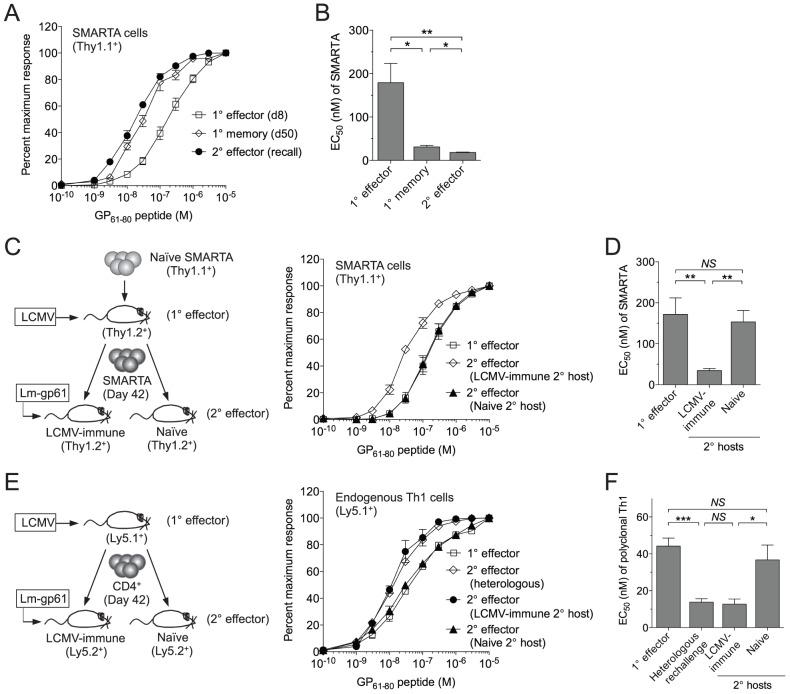
SMARTA memory cells lose functional avidity following heterologous rechallenge in naïve but not immune hosts. 1×10^4^ naïve SMARTA cells (Thy1.1^+^) were transferred into naïve B6 mice (Thy1.2^+^). *A*) At day 50 post-infection mice were given a heterologous rechallenge with Lm-gp61. Splenocytes were harvested 7 days later and restimulated with GP_61-80_ peptide *in vitro* for 4 hours in the presence of Brefeldin A, then permeabilized and stained for intracellular production of IFNγ. Curves display the functional avidity of SMARTA cells in the spleen at the peak of the primary response (day 8), following the establishment of memory (day 50) and 7 days after secondary challenge with Lm-gp61 (recall), as determined by the percent maximum frequency of IFNγ-producing SMARTA cells across the indicated range of peptide concentrations. *B*) Bar graph displays the effective peptide concentration required for a half maximal response (EC_50_), as determined by fitting the data to a sigmoidal curve (GraphPad Prism). *C*) At day 50 post-infection, we isolated and transferred 1×10^4^ SMARTA memory cells into naïve or LCMV-immune (day 50 LCMV infection-matched) secondary hosts, followed by Lm-gp61 secondary challenge and splenocyte harvest at day 7. Graph displays functional avidity of Thy1.1^+^ SMARTA cells at day 7 post-rechallenge. *D*) Bar graph indicates EC_50_ for Thy1.1^+^ SMARTA cells at day 7 post-rechallenge. *E*) We transferred 5×10^6^ CD4^+^ T cells from LCMV-immune mice (day 42, CD45.1^+^) into naïve or LCMV-immune secondary hosts (CD45.2^+^), followed by Lm-gp61 rechallenge. Graph shows functional avidity of CD4^+^CD45.1^+^ IFNγ-producing T cells in the spleen at the peak of the primary response, 7 days after rechallenge with Lm-gp61 (heterologous) or 7 days after rechallenge in naïve or LCMV-immune secondary hosts. *F*) Bar graph indicates EC_50_ for CD4^+^CD45.1^+^ IFNγ-producing T cells for each group. Error bars indicate the standard error of the mean (SEM) (n = 4–5 mice/group). Results are representative of three separate experiments. *p<.05; **p<.01; ***p<.001; *NS* = not significant, as determined by student's t-test.

We previously observed that both LCMV GP_61-80_-specific polyconal and TCR transgenic SMARTA Th1 cells undergo functional avidity maturation, as measured by their ability to make IFNγ in response to *ex vivo* restimulation with decreasing concentrations of GP_61-80_ peptide, during the transition from the Th1 effector phase to the development of long-lived memory [3]. To confirm this, we transferred small numbers of naïve SMARTA T cells (CD44^lo^, Thy1.1^+^) into naïve B6 hosts (Thy1.2^+^) and infected with LCMV one day later. SMARTA cells analyzed in the spleen following the establishment of memory (day 50) demonstrated higher functional avidity effector (day 8) SMARTA cells (Fig. 1A–B). Following heterologous rechallenge of LCMV-immune mice with recombinant Lm-gp61, secondary effector SMARTA cells continued to demonstrate high functional avidity (Fig. 1A–B).

Because SMARTA cells, a TCR monoclonal population, demonstrated remarkable plasticity in their ability to make a functional response to TCR restimulation, we sought to establish a model system in which we could better define the TCR-independent factors controlling the ability of these cells to translate TCR stimulation into a functional response. We employed a serial adoptive transfer system in which LCMV-induced SMARTA memory cells (Thy1.1^+^), generated as described above, underwent a second adoptive transfer into a naïve B6 secondary host (Thy1.1^+^) prior to Lm-gp61 rechallenge (Fig. 1C). Unlike what we observed following heterologous rechallenge, in this setting secondary SMARTA effector cells exhibited decreased functional avidity as compared to SMARTA memory cells prior to rechallenge (Fig. 1C–D).

One possible interpretation of these results is that the functional avidity of secondary SMARTA effector cells was influenced by differences in the inflammatory environment following heterologous rechallenge of LCMV-immune mice versus rechallenge of SMARTA memory cells in naïve mice. To test this, we adoptively transferred LCMV-induced SMARTA memory cells (Thy1.1^+^) into infection-matched LCMV-immune (>42 days post-infection) secondary recipients (Thy1.2^+^), followed by secondary stimulation with Lm-gp61 (Fig. 1C). SMARTA Th1 effector cells generated in LMCV-immune hosts exhibited high functional avidity at the peak of the secondary response (day 7), comparable to that of the originating SMARTA memory population (Fig. 1C-D). This observation was applicable to polyclonal T cell populations as well, as endogenous Th1 memory cells isolated from LCMV-immune mice and parked in naïve hosts also displayed a loss of functional avidity following secondary Lm-gp61 challenge, as compared to those parked in LCMV-immune secondary hosts (Fig. 1E–F). We concluded that functional avidity of secondary SMARTA cells was sensitive to extrinsic factors, potentially including the inflammatory environment and antigen load of the secondary challenge. One potential caveat to these assays is that differences in antigen presentation in naïve or LCMV-immune secondary hosts might impact measurements of functional avidity. However, in all cases, MHC Class II expression was similar or higher following challenge of naïve hosts, as compared to LCMV-immune hosts, and we observed no differences in the distribution or frequency of dendritic cells, macrophages or B cells (data not shown).

### The nature of the secondary challenge influences the size and stability of the secondary Th1 memory cells

**Figure 2:**
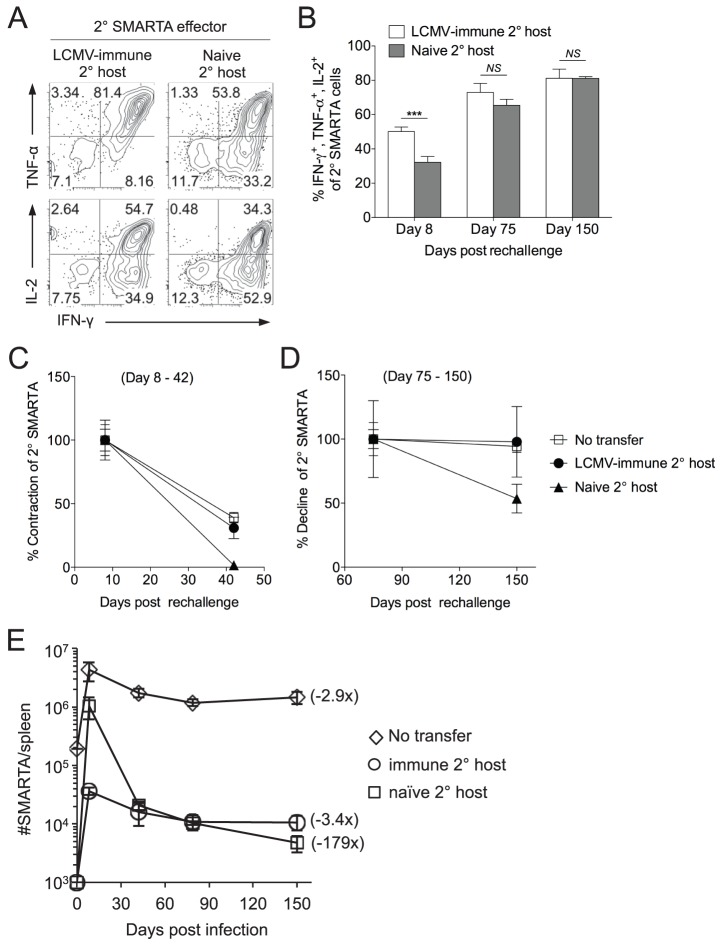
Secondary SMARTA effector cells generated after rechallenge in naïve hosts have decreased ability to produce multiple cytokines and limited memory differentiation potential. LCMV-induced memory SMARTA cells (>42 days post-infection) were transferred into either LCMV-immune or naïve hosts and rechallenged with Lm-gp61 as previously. *A*) Representative flow plots gated on Thy1.1^+^ SMARTA cells indicate the frequency of secondary SMARTA Th1 effector cells in the spleen of either LCMV-immune or naïve secondary hosts seven days after Lm-gp61 challenge that co-produced IFNγ and TNFα or IFNγ and IL-2 following *ex vivo* peptide restimulation. *B*) Bar graph indicates the frequency of Thy1.1^+^ secondary SMARTA effector (day 8) and memory (days 75, 150) cells in the spleen that simultaneously produced IFNγ, TNFα and IL-2 after *in vitro* peptide restimulation. *C*–*D*) Graphs display the percent contraction of the secondary SMARTA effector cells in the spleen between day 8 and 42 (C) and the percent decline of ensuing secondary memory cells between day 75 and 150 (D) after Lm-gp61 rechallenge of SMARTA memory cells (Thy1.1^+^) transferred into naïve or LCMV-immune secondary hosts (Thy1.2^+^), or of secondary SMARTA Th1 cells after rechallenge of LCMV-immune hosts without secondary transfer. E) Plot displays the total number of Thy1.1^+^ SMARTA cells in the spleen over a 150 day time course for each group. Numbers in parentheses indicate the fold decline in numbers between the peak of the secondary response (day 7) and day 150 post-rechallenge. Error bars indicate the SEM (n = 3–4 mice/group). Results are representative of two separate experiments. ***p<.001; *NS* = not significant, as determined by student's t-test.

In some settings, the generation of Th1 cells that can simultaneously produce multiple effector cytokines, particularly IFNγ, TNFα and IL-2 (“triple producers”), correlates to protective immunity to subsequent infections [33], [34]. Secondary SMARTA effector cells derived from challenge of naïve hosts showed a significant decline in the generation of triple cytokine producers at the peak of the secondary response, as compared to secondary SMARTA effector cells derived from challenge of LCMV-immune hosts, although these differences did not persist following the establishment of secondary memory (Fig. 2A–B). Rechallenge of SMARTA memory cells in naïve secondary hosts resulted in greater secondary expansion but exacerbated contraction and led to poor survival as secondary memory cells, as compared to rechallenge of SMARTA memory cells in LCMV-immune secondary hosts (Fig. 2C–D). The kinetics of secondary expansion, contraction and memory maintenance following rechallenge of SMARTA memory cells parked in LCMV-immune hosts replicated the kinetics of the secondary SMARTA response following heterologous Lm-gp61 rechallenge without a secondary transfer (Fig. 2E). On a per cell basis, secondary SMARTA Th1 effector cells generated following rechallenge in naïve hosts showed far less secondary memory potential. While the number of secondary SMARTA cells declined 3.4-fold between day 8 and day 150 following secondary stimulation after transfer into a LCMV-immune secondary host, and 2.9-fold following heterologous rechallenge without transfer, they declined 179-fold following secondary stimulation after transfer into a naive secondary host (Fig. 2C–E). Our findings confirm that the function, survival and memory potential of secondary Th1 effector cells are highly dependent on the environment induced by the secondary challenge.

Secondary SMARTA memory cells exhibited high functional avidity, regardless of whether SMARTA cells were rechallenged in naïve or LCMV-immune secondary hosts (Fig. S1). Additionally, tertiary challenge of secondary SMARTA memory cells derived from either group resulted in tertiary SMARTA Th1 effector cells with high functional avidity (Fig. S2). Therefore, the primary impact of differences in the secondary activation environment appeared to be difference in the number of long-lived secondary memory cells, not long-term differences in their functional capacity. An additional possibility was that our observations could apply only to a single boosting agent (Lm-gp61). This was not the case, as LCMV rechallenge of SMARTA memory cells transferred into naïve or Lm-gp61 host resulted in similar differences in the functional avidity of secondary SMARTA effector cells. (Fig. S3).

### Loss of functional avidity by secondary Th1 effector cells corresponds to increased magnitude and duration of secondary challenge

**Figure 3:**
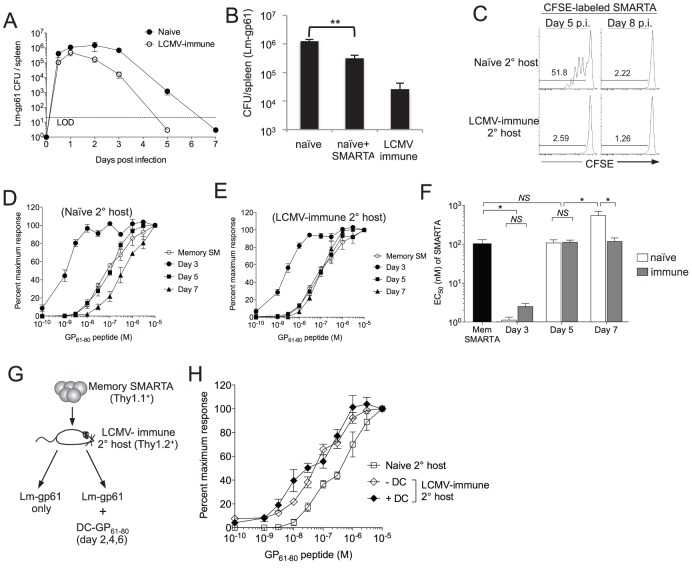
Functional avidity decay of secondary Th1 responders is associated with a prolonged infectious period. *A*) Graph displays the CFU detected in the spleen at the indicated time points after challenge of naïve or LCMV-immune mice with Lm-gp61. LOD is limit of detection. *B*) Bar graph indicates Lm-gp61 CFU in the spleen day 3 post-challenge of naïve mice, naïve mice that received a prior adoptive transfer of 2×10^5^ SMARTA memory cells and LCMV-immune mice. *C*) Naïve or LCMV-immune mice were infected with Lm-gp61. At either day 5 or 8 post-infection, they were injected with CFSE-labeled naïve SMARTA cells (Thy1.1^+^). Representative flow plots indicate CFSE dilution by gated CD4^+^Thy1.1^+^ SMARTA cells in the spleen 3 days later. *D–F*) 1×10^5^ LCMV-induced memory SMARTA cells (Thy1.1^+^, >42 days post-infection) were transferred into either naïve or LCMV-immune secondary hosts (Thy1.2^+^) and rechallenged with Lm-gp61. The functional avidity of Thy1.1^+^ SMARTA cells was determined as previously at days 3, 5 and 7 post-infection in naive (D) or LCMV-immune (E) secondary hosts. *F*) Bar graph displays EC_50_. *G*) 1×10^4^ Thy1.1^+^ LCMV-induced memory SMARTA cells (>42 days post-infection) were transferred into LCMV-immune or naïve B6 mice (Thy1.2^+^), followed by Lm-gp61 challenge one day later, with or without co-immunization with 1×10^6^ peptide-loaded DCs on days 2, 4 and post-challenge. *H*) Graph displays the functional avidity of secondary SMARTA effector cells in the spleen at day 7 after Lm-gp61 challenge of LCMV-immune secondary hosts with or without DC co-immunization. Error bars indicate the SEM (n = 3–4 mice/group). Results are representative of two separate experiments. *p<.05; *NS* = not significant, as determined by student's t-test.

To better understand the infectious environment following re-activation of SMARTA memory cells parked in either naïve or LCMV-immune hosts, we investigated the kinetics of pathogen clearance and antigen presentation in each setting. During the course of the Lm-gp61 challenge, LCMV-immune mice exhibited more rapid clearance kinetics and significantly lower bacterial loads starting at day 3, as compared to challenge of naïve mice (Fig. 3A). Rapid clearance kinetics may have been due to the direct contribution of Th1-mediated secondary immunity or CTL-mediated immunity to a previously described Class I-restricted epitope within GP_61-80_ [35]. Therefore, we transferred 2×10^5^ SMARTA memory cells into naïve mice prior to Lm-gp61 infection. Prior transfer of SMARTA memory cells led to a ∼4-fold decrease in colony forming units (CFU) in the spleen by day 3 post-challenge (Fig. 3B), indicating a direct protective role for CD4^+^ memory T cells in this model. Antigen presentation was undetectable by day 5 after Lm-gp61 challenge of LCMV-immune mice, whereas antigen presentation was still readily detectable following challenge of naïve mice (Fig. 3C).

We further sought to determine whether changes in functional avidity were associated with functional plasticity in individual cells or merely the selective outgrowth of high or low functional avidity effector cells under distinct restimulation conditions. We assessed the kinetics of changes in functional avidity throughout the secondary response. At day 3 post-rechallenge, secondary SMARTA effector cells derived from rechallenge of both naïve and LCMV-immune hosts exhibited a massive increase in functional avidity, requiring ∼50-fold lower peptide concentration to induce a half-maximal response (Fig. 3D–F). By day 5 post-rechallenge, the functional avidity of secondary SMARTA Th1 effector cells in both groups had declined, but only secondary SMARTA Th1 effector cells generated after rechallenge in naïve hosts underwent a continued loss in functional avidity, with a further 5-fold reduction in antigen sensitivity by day 7 post-infection correlating to the period of time in which the secondary challenge persists in these mice (Fig. 3D–F). Our findings indicate that secondary SMARTA effector cells maintain the capacity for remarkable functional plasticity in a manner dependent on their rechallenge environment.

Lastly, we determined whether extended antigen presentation in the later stages of the secondary response was sufficient to induce a loss of functional avidity by secondary CD4^+^ effector cell. We parked SMARTA memory cells in LCMV-immune hosts, challenged with Lm-gp61 as above and co-immunized with GP_61-80_ peptide-loaded DCs at days 2, 4 and 6. Secondary SMARTA effector cells maintained high functional avidity regardless of DC co-immunization, indicating that extending the period of antigen presentation alone had no impact on secondary SMARTA functional development (Fig. 3G-H).

### Functional avidity of secondary CD4^+^ effector T cells is not regulated by access to antigen

**Figure 4:**
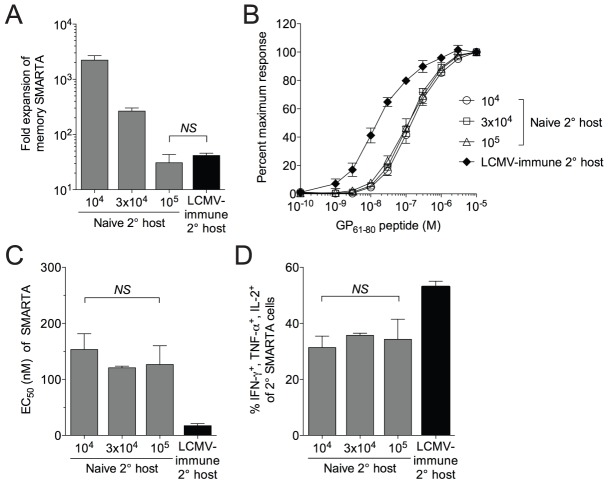
Functional avidity decay of memory SMARTA cells restimulated in naïve secondary hosts is independent of the magnitude and duration of antigen availability. *A*) We transferred 1×10^4^, 3×10^4^ or 1×10^5^ LCMV-induced SMARTA memory cells (Thy1.1^+^, >42 days post-infection with LCMV) into naïve B6 secondary hosts (Thy1.2^+^) followed by challenge with Lm-gp61. Estimating a 10% “take” of transferred cells, the bar graph indicates the fold secondary expansion of each population of SMARTA cells in the spleen by day 7 post-infection, as compared to SMARTA cells (Thy1.1^+^, 1×10^4^ transferred) rechallenged in LCMV-immune hosts. *B*) Graph indicates the functional avidity of secondary SMARTA Th1 effector cells in the spleen day 7 after transfer of the indicated numbers into naïve B6 secondary hosts and challenge with Lm-gp61, or day 7 after transfer of 1×10^4^ SMARTA cells into LCMV-immune secondary hosts and challenge with Lm-g61. *C*) Bar graph indicates the EC_50_ of each group of SMARTA cells, calculated as previously. *D*) Bar graph indicates the frequency of Thy1.1^+^ SMARTA cells in the spleen for each group the simultaneously produce IFNγ, TNFα and IL-2. Error bars indicate the SEM (n = 4 mice/group). *NS* = not significant, as determined by student's t-test.

Various factors may influence the functionality of secondary effector, including competition with other Th1 cells for antigen and resources, differences in priming, alterations in the inflammatory cytokine environment and the duration of the secondary challenge. We tested the hypothesis that functional avidity of secondary SMARTA effector cells might be influenced by access to antigen. CD4^+^ T cells are particularly sensitive to inter- and intraclonal competition for antigen [36], [37]. To generate a system for limiting access to antigen *in vivo*, we transferred increasing numbers of SMARTA memory cells into naïve secondary hosts prior to Lm-gp61 challenge. As expected, while transfer of 1×10^4^ SMARTA memory cells resulted in robust secondary expansion, transfer of 10-fold higher numbers of SMARTA memory cells (1×10^5^) resulted in sharply decreased secondary expansion that was similar to the magnitude of secondary SMARTA cell expansion in LCMV-immune hosts (Fig. 4A). However, both changes to functional avidity as well as the ability to produce cytokines following *in vitro* restimulation were independent of clonal expansion (Fig. 4B-D). Therefore, we concluded that functional differentiation of secondary SMARTA Th1 effector cells was not dependent on increased access to antigen-dependent activation and differentiation signals.

### Functional avidity of secondary effector T cells is controlled by the inflammatory environment

**Figure 5:**
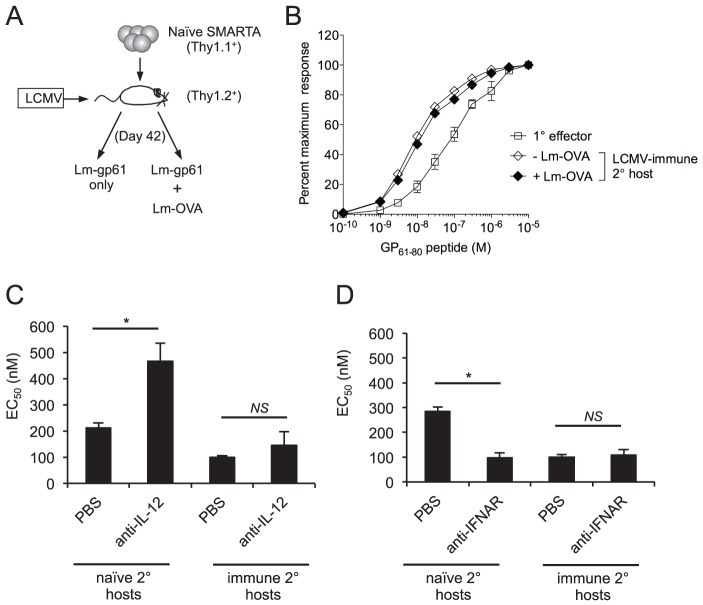
Functional avidity of secondary SMARTA Th1 cells is regulated by inflammatory environment. *A*) Memory SMARTA cells (Thy1.1^+^) in LCMV-immune hosts (Thy1.2^+^, >42 days post-infection) were rechallenged with Lm-gp61 alone or co-challenged with Lm-gp61 and Lm-OVA. *B*) Graph displays the functional avidity of secondary SMARTA effectors cells in the spleen at day 7 after Lm-gp61 rechallenge with or without Lm-Ova co-immunization, as compared to primary SMARTA Th1 effector cells in the spleen 8 days after LCMV infection. *C*) Graph displays the EC_50_ of secondary SMARTA Th1 cells in the spleen after rechallenge in naïve or LCMV-immune secondary hosts. Indicated groups of mice were treated with anti-IL-12 neutralizing antibody or PBS prior to challenge. *D*) Graph displays the EC_50_ of secondary SMARTA effector cells in the spleen day 7 after rechallenge in naïve or LCMV-immune secondary hosts. Indicated groups of mice were treated with anti-IFNAR1 antibody or PBS prior to challenge. Error bars indicate the SEM (n = 4 mice/group). *p<.05; *NS* = not significant, as determined by student's t-test.

We considered two possibilities that might account for the role of pathogen-dependent inflammation in the control of T cell functional avidity. First, extended exposure to inflammation during Lm-gp61 stimulation of SMARTA cells in naïve hosts might lead to a decrease in T cell functional avidity. Second, qualitative or quantitative differences in in the inflammatory cytokine milieu following Lm-gp61 challenge of naïve or LCMV-immune hosts could account for differences in functional avidity. To test the possibility that extending the inflammatory environment alone could modulate the functional avidity of secondary Th1 cells, we co-challenged LCMV-immune mice containing SMARTA memory cells with Lm-gp61 and a recombinant *Listeria* expressing the irrelevant antigen OVA (Lm-OVA), thus inducing longer lasting *Listeria* infection but without extending the time frame of GP_61-80_ antigen presentation. Due to the fact that Lm-Ova is erythromycin resistant, we were able to measure the colony forming units (CFU) in the spleen for both Lm-gp61 and Lm-Ova following co-challenge. Both Lm-gp61 and Lm-OVA reached similar bacterial loads by day 3 post-infection, but at day 5, when Lm-gp61 was undetectable in the spleen, Lm-Ova persisted at levels similar to the bacterial burden observed in naïve mice infected with Lm-gp61 alone (data not shown). Extending duration of infection-induced inflammation following heterologous challenge was not sufficient to induce a loss of functional avidity by secondary SMARTA effector cells (Fig. 5A–B). Similarly, clearance of Lm-gp61 mediated by ampicillin treatment 24 or 48 hours after SMARTA rechallenge in naïve hosts had no impact on their functional avidity at the peak of the secondary response (data not shown). Collectively, these findings demonstrate that the duration of the inflammatory response does not by itself significantly influence secondary effector T cell functional avidity.

We further tested whether perturbations in the inflammatory cytokine milieu might influence the functional avidity of secondary CD4^+^ effector T cells. We targeted two key inflammatory pathways by treating mice with anti-IL-12 neutralizing antibodies or blocking antibodies to IFN α/β receptor 1 (IFNAR1) following SMARTA cell rechallenge in either naïve or LCMV-immune hosts. Antibody treatment did not significantly influence Lm-gp61 bacterial load at day 3 post-rechallenge (data not shown), a finding that could be due to the partially attenuated nature of Lm-gp61. Following SMARTA rechallenge in naïve hosts, IL-12 neutralization resulted in poor secondary effector cell functional avidity (Fig. 5C), whereas IFNAR1 blockade resulted in a significant increase in secondary effector cell functional avidity (Fig 5D). Neither IL-12 neutralization nor IFNAR1 blockade had any effect on functional avidity following SMARTA rechallenge in immune hosts (Fig. 5C–D). Further studies are necessary to determine whether antibody-mediated changes to the inflammatory milieu or the direct action of these cytokines accounted for differences in the acquisition of functional avidity by secondary SMARTA Th1 effector cells. However, these results overall indicate a clear role for the inflammatory environment in controlling the functional differentiation of secondary CD4^+^ effector T cells.

### Loss of high antigen sensitivity corresponds to differential TCR signaling

**Figure 6:**
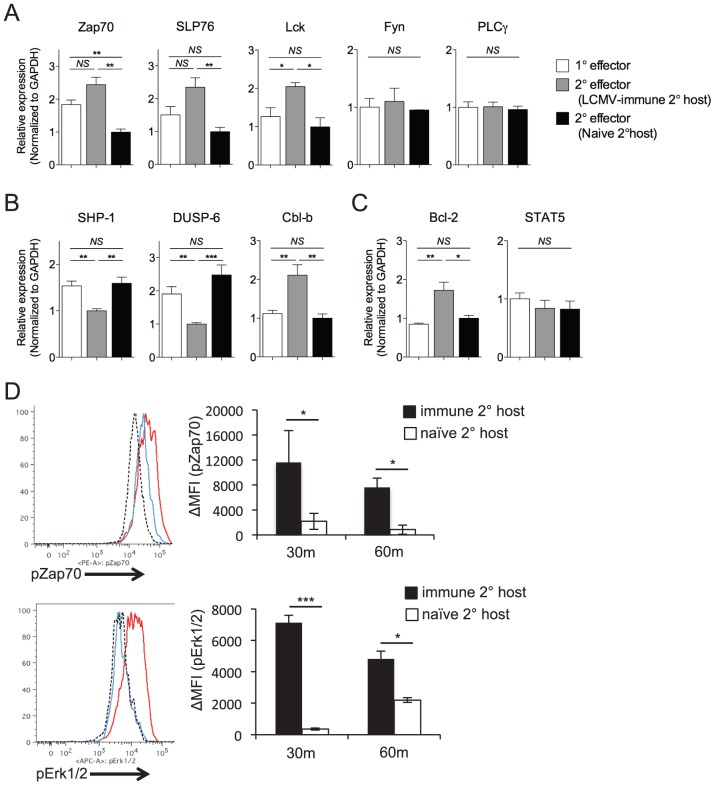
Maintenance of high functional avidity after secondary challenge is associated with enhanced expression of TCR signaling molecules. The relative gene expression of *A*) Zap70, SLP76, Lck, Fyn, and PLCγ; *B*) SHP-1, DUSP-6, Cbl-b; and *C*) Bcl-2 and STAT5 were evaluated by quantitative RT-PCR of FACS-sorted Thy1.1^+^ secondary SMARTA effector cells in the spleen 7 days after Lm-gp61 challenge of LCMV-immune or naïve secondary hosts, or of primary SMARTA effector cells 8 days after LCMV infection. Results were normalized to GAPDH. *D*) Representative flow plots indicate pZap70 and pErk1/2 expression by SMARTA cells after 30 minutes of in vitro peptide restimulation. Histograms indicate secondary SMARTA effector cells (day 7) after Lm-gp61 rechallenge in naïve (blue) or LCMV-immune (red) secondary hosts, as compared to unstimulated controls (dashed). Bar graphs indicate the change in mean fluorescence intensity (MFI) following 30 or 60 minutes of peptide restimulation. Error bars indicate the SEM (n = 4 mice/group). Results are representative of two separate experiments. *p<.05; **p<.01; ***p<.001; *NS* = not significant, as determined by student's t-test.

To determine whether loss of functional avidity was associated with specific defects in the ability of secondary Th1 effector cells to initiate TCR-dependent signaling events, we analyzed differences in gene expression levels of TCR signaling molecules, survival factors, and signaling regulators. We observed enhanced gene expression of some (Zap70, Lck, and SLP76) but not all (Fyn, PLCγ) proximal TCR signaling molecules by SMARTA cells rechallenged in LCMV-immune hosts, as compared to those rechallenged in naïve hosts (Fig. 6A). Conversely, SMARTA cells rechallenged in naïve hosts displayed increased expression of two molecules (SHP-1, DUSP-6) known to dampen TCR-dependent kinase activity (Fig. 6B)[38]–[42]. Again, this was not universally true, as SMARTA cells rechallenged in naïve hosts demonstrated decreased expression of Cbl-b (Fig. 6B), an anergy-associated E3 ubiquitin ligase that regulates T cell activation by targeting proximal TCR signals [43], [44]. Secondary SMARTA effector cells derived from challenge of LCMV-immune mice exhibited increased gene expression of Bcl-2, a well-known CD4^+^ T cell survival factor, while demonstrating no difference in STAT5 expression, a transcription factor upstream of several pro-survival pathways (Fig. 6C). Overall, the expression profile of SMARTA recall responses in LCMV-immune secondary hosts partially skewed towards pro-survival and pro-TCR signaling. To determine the effect on TCR signaling, we briefly restimulated secondary SMARTA effector cells with GP_61-80_ peptide *in vitro*. In accordance with the gene expression levels, secondary SMARTA recall responses in naive hosts exhibited a decreased ability to induce TCR signaling events, demonstrating reduced phosphorylation of Zap-70 and Erk1/2 following *in vitro* peptide restimulation for 30 or 60 minutes (Fig. 6D). These findings suggest that differences in functional avidity, as determined by IFNγ production, are linked to differences in the ability to mediate TCR signaling events.

## Discussion

We employed two adoptive transfer systems to investigate the impact of the infectious environment in the expansion, function and survival of secondary effector and memory CD4^+^ T cell responses. LCMV-induced SMARTA memory cells were transferred into LCMV-immune or naïve secondary hosts and then rechallenged with Lm-gp61. Due to differences in pathogen clearance between these two model systems, we explored the relative contributions of antigen and inflammation to secondary effector differentiation and the development of long-lived secondary memory. Based on our studies, we conclude that the inflammatory context of the rechallenge has profound consequences for secondary CD4^+^ effector and memory T cell differentiation. Rechallenge of SMARTA memory cells in LCMV-immune secondary hosts resulted in secondary responses with high functional avidity, limited secondary contraction and stable maintenance within the memory pool. The kinetics of the secondary response was similar to that seen following heterologous rechallenge. In contrast, rechallenge of SMARTA cells in naïve secondary hosts resulted in secondary responses with poor functional avidity, severe contraction and decline within the memory pool.

A key biological consequence of rechallenge in these two settings is the number of resulting memory cells. While small numbers of SMARTA memory cells rechallenged in naïve secondary hosts enjoyed a proliferative advantage over those rechallenged in LCMV-immune secondary hosts, this advantage disappeared when SMARTA memory cells were present at more physiologically relevant levels. Furthermore, their numerical advantage during the secondary effector response was lost due to their severe contraction and poor survival within the memory pool. Rechallenge within the immune environment led to better boosting of memory cell numbers. Because we show a direct protective role for CD4^+^ memory T cell following *Listeria* challenge, the overall numbers of memory cells are likely to be a key measure of the efficacy of vaccination and immunotherapeutic strategies aimed at stimulating CD4^+^ T cell-mediated protection.

Our report here corresponds to our previous studies linking functional avidity at the peak of the effector response to subsequent memory potential [3], [31]. Therefore, we propose that identifying the factors that promote high functional avidity during the effector response will be a key step toward understanding memory differentiation. While the functional avidity of secondary effector T cells was independent of access to antigen, we found a key role for the inflammatory environment, as disruption of the IL-12 or IFN-I inflammatory pathways led to a decrease or increase, respectively, of secondary effector cell functional avidity. The role of inflammatory cytokines in controlling T cell function and protective capacity is complex, particularly with regard to the roles of IL-12 and IFN-I. While IFN-I has long been studied as a key factor in restricting viral replication, disruption of this inflammatory pathway enhances *Listeria* clearance [45], [46]. A recent study also found that IFNAR1 blockade resulted in enhanced control of chronic viral infection in a CD4^+^ T cell-dependent manner [47]. In contrast, T cell-intrinsic IFN-I signaling promotes their expansion [48], [49]. Recent findings have focused on the importance of inflammatory cytokines, including IFN-I, IL-12, and IL-18, in promoting the increased antigen sensitivity of local effector primary and secondary CD8^+^ T cells independent of antigen or clonal selection [24], [25]. One possible hypothesis resulting from our studies is that IFN-I and IL-12 have opposing effects in controlling the functional avidity of CD4^+^ effector T cells. However, future studies are required to distinguish whether antibody-mediated disruption of IL-12 and IFN-I in the present report reflect a T cell intrinsic influence of IFN-I and/or IL-12-mediated signaling on secondary effector T cell function and subsequent memory potential or an indirect influence on secondary effector T cell function due to changes in the inflammatory milieu.

While it is well established that the context of the primary infection is important for the differentiation, stability, and functional maturation of effector Th1 cells [2], [50], our findings show that the context of the secondary challenge can have profound consequences for the functional maturation of responding secondary Th1 effector cells and the long-term survival of subsequent Th1 memory populations. Functional attributes are not permanently imprinted on Th1 cells during primary activation. Instead, secondary Th1 differentiation demonstrates functional plasticity that is dependent on the context of the secondary challenge. Secondary SMARTA Th1 effector cells showed remarkable plasticity throughout secondary response. We have previously shown that in the context of a homologous rechallenge, where memory Th1 cells are weakly stimulated due to the limited persistence of the infection, secondary effector Th1 cells exhibit decreased functional avidity and diminished long-term survival [31]. A key conclusion of these studies is that secondary Th1 effector and memory differentiation are acutely sensitive to the context of the secondary challenge. We propose that more precise identification of environmental signals that enhance the function and memory potential of secondary Th1 effector cells will allow more effective design of boosting and immunotherapeutic strategies designed to optimize T cell function, protective capacity and long-term survival in high numbers.

We recently reported an important role for sustained interactions between TCR and antigen in promoting CD4^+^ memory T cell differentiation [4]. One intriguing hypothesis is that non-specific differentiation cues, such as those delivered by inflammatory cytokines, could influence TCR signaling sensitivity and subsequent TCR-dependent memory differentiation. Other studies have shown that enhanced antigen sensitivity by T cells correlates to up-regulated expression of proximal TCR signaling molecules [51], [52]. Their induction is TCR-dependent in other settings [39], [40], [42], and while their up-regulation may represent, at least in part, antigen-driven feedback in regulating the ongoing secondary response, another likely conclusion is that their activity is regulated by environmental inflammatory cues. Our finding that differences in early TCR signaling events are associated with inflammation-dependent differences in functional avidity supports this idea. Future studies are required to understand the interplay between environmental and TCR-driven differentiation cues in the formation of primary and secondary memory T cells.

## Materials and Methods

### Ethics statement

This study was carried out in accordance with the recommendations provided by the Guide for the Care and Use of Laboratory Animals of the National Institutes of Health. This study was approved by the University of Utah Animal Care and Use Committee (PHS Assurance Registration Number A3031-01, Protocol Number 12-10011).

### Mice and infections

6-8 week old C57BL/6 (B6) mice were purchased from The Jackson Laboratory. SMARTA TCR transgenic mice [53] were maintained at the University of Utah. Lymphocytic choriomeningitis virus (LCMV) Armstrong 53b was grown in BHK cells and titered in Vero cells [54]. Mice were infected i.p. with 2×10^5^ plaque-forming units (PFU). *Listeria monocytogenes* expressing the GP_61-80_ epitope of LCMV (Lm-gp61, M. Kaja-Krishna, University of Washington) and *Listeria monocytogenes* expressing OVA (Lm-OVA) were propagated in BHI broth and agar plates. Prior to infection, the bacteria were grown to log phase and concentration was determined by measuring the O.D. at 600 nm (O.D. of 1 = 1×10^9^ CFU/ml). For primary infections or secondary rechallenge of LCMV-immune mice (>42 days post-infection), mice were injected i.v. with 2×10^5^ CFU Lm-gp61. For Lm-OVA, mice were injected i.v. with 1×10^4^ CFU.

### Adoptive transfers and antibody treatments

To generate primary SMARTA memory cells, untouched CD4^+^ T cells were isolated from the spleens of SMARTA mice (Thy1.1^+^) using a MACS CD4^+^ T cell isolation kit (Miltenyi Biotec). In addition, we added biotinylated anti-CD44 antibody (eBiosciences, San Diego, CA) to eliminate CD44^hi^ “memory phenotype” SMARTA as previously [3]. Naïve SMARTA cells were re-suspended in PBS and injected i.v. into recipient mice (Thy1.2^+^) 1 day prior to LCMV infection. For adoptive transfer of memory SMARTA cells, CD4^+^ T cells were isolated from the spleens of LCMV-immune B6 mice containing memory SMARTA cells (>day 42 post-infection) and then injected i.v. into secondary recipients that were subsequently infected 1 day later. Similarly, for adoptive transfer of endogenous GP_61-80_-specific Th1 memory cells, CD4^+^ T cells were enriched from the spleens of LCMV-immune B6 mice (>d42 days post infection), and 5×10^6^ CD4^+^ T cells were injected i.v. into secondary recipients prior to rechallenge. For anti-IL-12 antibody treatments, mice received a 0.5 mg injection of anti-IL-12 antibody (clone C17.8) one day prior to challenge, as previously [55], [56]. For IFN-I blockade, mice received a 1.25 mg injection of anti-IFNAR1 antibody (clone MAR1-5A3) i.p. one day prior to infection as previously [47], [57].

### Dendritic cell immunizations

DCs were expanded in B6 mice with a Flt-3L-secreting B16 mouse melanoma cell line as previously described [30], [58]. DCs were enriched to 70–80% purity from the spleens and lymph nodes by transient adherence overnight. They were then pulsed with 1 µM LCMV GP_61-80_ peptide for 2h in the presence of 1 µg/ml LPS. LCMV-immune mice (>d42 days post infection) were rechallenged with Lm-gp61 and subsequently injected with 1×10^6^ DCs i.v. on days 2, 4, and 6 post-infection.

### Cell preparations and flow cytometry

Splenocytes were placed in single-cell suspension in DMEM containing 10% FBS and supplemented with antibiotics and L-glutamine. For CFSE experiments, naïve SMARTA splenocytes were labeled using the CellTrace CFSE Labeling Kit (Invitrogen), according to the manufacturer's instructions, followed by i.v. transfer (1×10^6^ SMARTA/mouse). For cell surface staining, cells were incubated with fluorescent dye-conjugated antibodies, with specificities as indicated (eBiosciences, San Diego, CA, or BD Biosciences, Mountain View, CA), in PBS containing 1% FBS. Antibody-stained cells were detected on a FACSCanto II flow cytometer (BD Biosciences) and results were analyzed using FlowJo software (TreeStar).

### Peptide restimulation and intracellular staining

Re-suspended cells were restimulated for 4 h with 10 µM GP_61–80_ peptide (GLKGPDIYKGVYQFKSVEFD) in the presence of brefeldin A (GolgiPlug, 1 µl/ml). Cells were stained with cell surface Abs, permeabilized and stained with cytokine specific antibodies using a kit, per the manufacturer's instructions (BD Biosciences). For functional avidity assays, cells were restimulated with a range of peptide concentrations (10 µM–0.1 nM) prior to cytokine staining, with the percentage of maximal response determined by calculating the frequency of IFNγ–producing cells at any given concentration as a percentage of the frequency of IFNγ–producing cells at the highest peptide concentration. For intracellular staining of phosphorylated Erk1/2 (T204/Y202)(eBioscience) and Zap70 (Y319/Y352)(BDBiosciences), mice were restimulated with 10 µM peptide for 30 or 60 minutes, followed by intracellular phospho-staining using a kit per the manufacturer's instructions (BD Biosciences).

### RNA isolation and RT-PCR

Total RNA was isolated using the RNeasy kit (Qiagen, Valencia, CA) from FACS-sorted primary SMARTA effectors and secondary SMARTA effectors induced in either LCMV-immune or naïve hosts. cDNA was prepared from the RNA and real-time RT-PCR was performed on a Roche LightCycler 480 (Roche, Indianapolis, IN) using Superscript III Platinum Two-Step qRT-PCR Kit with SYBR Green (Invitrogen, Carlsbad, CA), according to the manufacturer's instructions. Expression levels were normalized to GAPDH expression. Oligonucleotide primer sets used are as follows: Zap70: F-AGCGAATGCCCTGGTATCAC, R-CCAGAGCGTGTCAAACTTGGT; SLP76: F-AGAATGTCCCGTTTCGCTCAG, R-TGCTCCTTCTCTCTTCGTTCTT; Lck: F-TGGTCACCTATGAGGGATCTCT, R-CGAAGTTGAAGGGAATGAAGCC; Fyn: F-ACCTCCATCCCGAACTACAAC, R-CGCCACAAACAGTGTCACTC; PLCγ: F-ATCCAGCAGTCCTAGAGCCTG, R-GGATGGCGATCTGACAAGC; SHP-1: F-CCCGCTCAGGGTCACTCATA, R-CCCGAGTAGCGTAGTAAGGCT; DUSP-6: F-CCGTGGTGCTGTACGACGAG, R-GCAGTGCAGGGCGAACTCGGC; Cbl-b: F-GTCGCAGGACAGACGGAATC, R-GAGCTGATCTGATGGACCTCA; Bcl-2: F-GTGGTGGAGGAACTCTTCAGGGATG, R-GGTCTTCAGAGACAGCCAGGAGAAATC; STAT5A: F-CGCCAGATGCAAGTGTTGTAT, R-TCCTGGGGATTATCCAAGTCAAT; GAPDH: F-ATTGTCAGCAATGCATCCTG, R-ATGGACTGTGGTCATGAGCC.

## Supporting Information

Figure S1 Secondary SMARTA memory cells exhibit high functional avidity regardless of the environment of the secondary challenge. LCMV-induced SMARTA memory cells (>42 days post-infection, Thy1.1^+^) were transferred into naïve or LCMV-immune (>42 days post-infection) secondary hosts (Thy1.2^+^), followed by challenge with Lm-gp61, as in Fig. 1. Graphs display the functional avidity and EC_50_ of secondary SMARTA effector (day 8) and memory (day 42, 75) cells following rechallenge in naïve (A–B) or LCMV-immune (C–D) secondary hosts. Functional avidity was plotted as the percent of the maximal frequency of SMARTA IFNγ-producers for each peptide concentration. EC_50_ was calculated by fitting the data to a sigmoidal curve (GraphPad Prism). Error bars indicate SEM (n = 4 mice/group). *p<.05; **p<.01; *NS* = not significant, as determined by student's t-test.
(PDF)

Figure S2 Tertiary SMARTA effector cells acquire high functional avidity following heterologous tertiary challenge. *A*) LCMV-induced SMARTA memory cells (>42 days post-infection Thy1.1^+^) were transferred into naïve secondary hosts (Thy1.2^+^), followed by challenge with LCMV to induce the generation of secondary SMARTA effector and memory cells. Upon development of secondary memory (42 days post-infection), mice were given a heterologous rechallenge with Lm-gp61. *B*) Functional avidity dose response curves were generated for secondary SMARTA effector cells (day 8 after LCMV challenge), secondary memory SMARTA cells (day 42 after LCMV challenge) and tertiary effector SMARTA cells (day 8 after Lm-gp61 heterologus rechallenge) in the spleen. *C*) Bar graphs indicates the EC_50_ of SMARTA cells for each group. Error bars indicate SEM (n = 4 mice/group). ****p<.0001; *NS* = not significant, as determined by student's t-test.
(PDF)

Figure S3 Secondary SMARTA memory parked in naive secondary hosts undergo functional avidity decay upon LCMV challenge. *A*) LCMV-induced SMARTA memory cells (>42 days post-infection, Thy1.1^+^), generated as previously, were transferred into naïve or Lm-gp61-immune (>42 days post-infection) secondary hosts (Thy1.2^+^), followed by challenge with LCMV. *B*) Functional avidity dose response curves were generated for secondary SMARTA effector cells (day 8) in the spleens of naïve and Lm-gp61-immune secondary hosts, as previously. Error bars indicate SEM (n = 4 mice/group).
(PDF)
