# Supplementary figures and images for: Dynamic Functional Modulation of CD4+ T Cell Recall Responses Is Dependent on the Inflammatory Environment of the Secondary Stimulus
Source: PLoS Pathog. 2014 May 22;10(5):e1004137. doi: 10.1371/journal.ppat.1004137 (PMC4031222; doi:10.1371/journal.ppat.1004137)

Figure S1

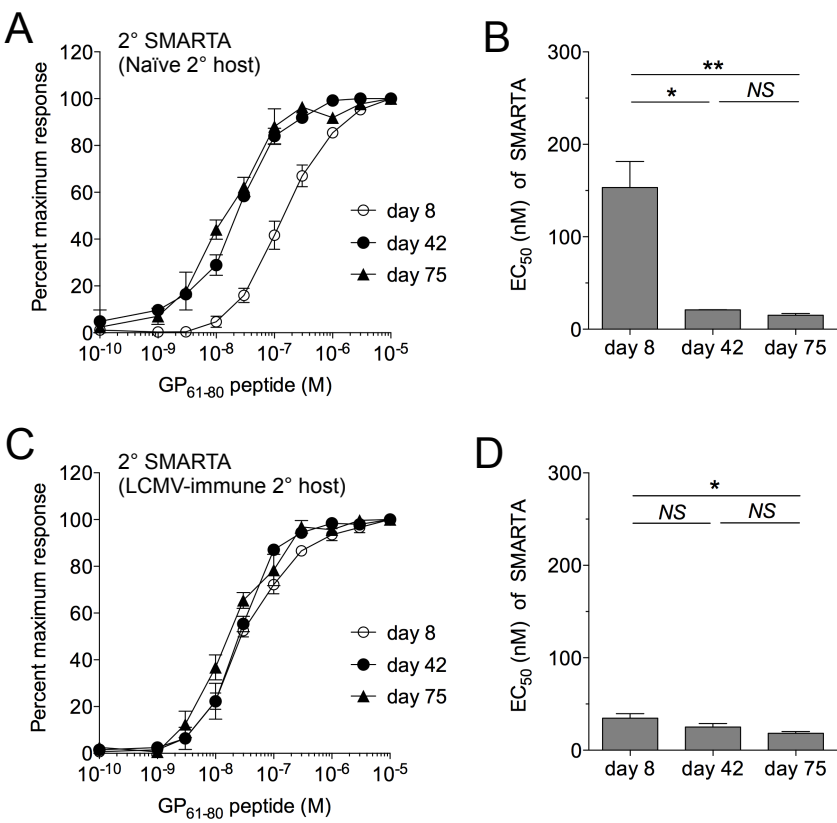

Supplement: Figure S1 — Secondary SMARTA memory cells exhibit high functional avidity regardless of the environment of the secondary challenge. LCMV-induced SMARTA memory cells (>42 days post-infection, Thy1.1+) were transferred into naïve or LCMV-immune (>42 days post-infection) secondary hosts (Thy1.2+), followed by challenge with Lm-gp61, as in Fig. 1. Graphs display the functional avidity and EC50 of secondary SMARTA effector (day 8) and memory (day 42, 75) cells following rechallenge in naïve (A–B) or LCMV-immune (C–D) secondary hosts. Functional avidity was plotted as the percent of the maximal frequency of SMARTA IFNγ-producers for each peptide concentration. EC50 was calculated by fitting the data to a sigmoidal curve (GraphPad Prism). Error bars indicate SEM (n = 4 mice/group). *p<.05; **p<.01; NS = not significant, as determined by student's t-test. (PDF) [file ppat.1004137.s001.pdf]

Figure S2

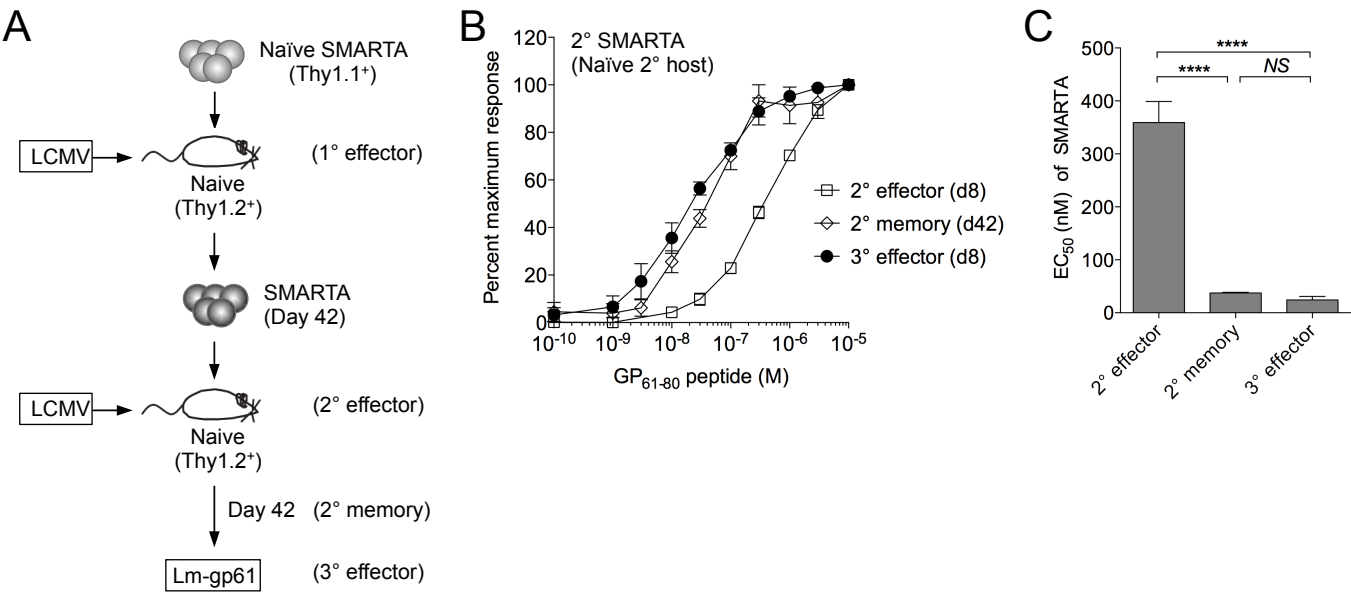

Supplement: Figure S2 — Tertiary SMARTA effector cells acquire high functional avidity following heterologous tertiary challenge. A) LCMV-induced SMARTA memory cells (>42 days post-infection Thy1.1+) were transferred into naïve secondary hosts (Thy1.2+), followed by challenge with LCMV to induce the generation of secondary SMARTA effector and memory cells. Upon development of secondary memory (42 days post-infection), mice were given a heterologous rechallenge with Lm-gp61. B) Functional avidity dose response curves were generated for secondary SMARTA effector cells (day 8 after LCMV challenge), secondary memory SMARTA cells (day 42 after LCMV challenge) and tertiary effector SMARTA cells (day 8 after Lm-gp61 heterologus rechallenge) in the spleen. C) Bar graphs indicates the EC50 of SMARTA cells for each group. Error bars indicate SEM (n = 4 mice/group). ****p<.0001; NS = not significant, as determined by student's t-test. (PDF) [file ppat.1004137.s002.pdf]

Figure S3

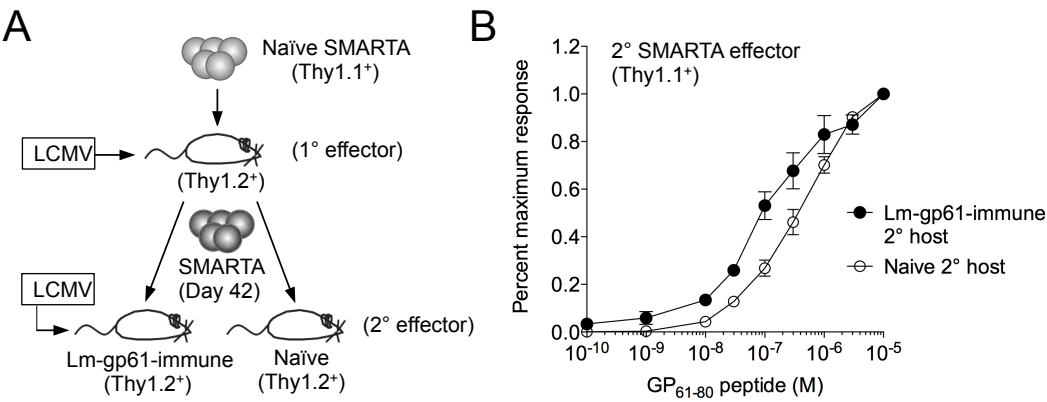

Supplement: Figure S3 — Secondary SMARTA memory parked in naive secondary hosts undergo functional avidity decay upon LCMV challenge. A) LCMV-induced SMARTA memory cells (>42 days post-infection, Thy1.1+), generated as previously, were transferred into naïve or Lm-gp61-immune (>42 days post-infection) secondary hosts (Thy1.2+), followed by challenge with LCMV. B) Functional avidity dose response curves were generated for secondary SMARTA effector cells (day 8) in the spleens of naïve and Lm-gp61-immune secondary hosts, as previously. Error bars indicate SEM (n = 4 mice/group). (PDF) [file ppat.1004137.s003.pdf]
